# Polyunsaturated Fatty Acids and Glycemic Control in Type 2 Diabetes

**DOI:** 10.3390/nu11051067

**Published:** 2019-05-14

**Authors:** Vibeke H. Telle-Hansen, Line Gaundal, Mari C.W. Myhrstad

**Affiliations:** Faculty of Health Sciences, Oslo Metropolitan University, Postbox 4, St. Olavsplass, 0130 Oslo, Norway; vtelle@oslomet.no (V.H.T.-H.); linega@oslomet.no (L.G.)

**Keywords:** PUFA, polyunsaturated fatty acids, glycemic control, nuts, fish, fish oil, vegetable oil, type 2 diabetes

## Abstract

The impact of dietary fat on the risk of cardiovascular disease (CVD) has been extensively studied in recent decades. Solid evidence indicates that replacing saturated fatty acids (SFAs) with polyunsaturated fatty acids (PUFAs) decreases blood cholesterol levels and prevents CVD and CVD mortality. Studies indicate that fat quality also may affect insulin sensitivity and hence, the risk of type 2 diabetes (T2D). A high intake of SFAs has shown to increase the risk of T2D in prospective studies, while a high intake of PUFAs reduces the risk. Whether PUFAs from marine or vegetable sources affect glycemic regulation differently in T2D remains to be elucidated. The aim of the present review was therefore to summarize research on human randomized, controlled intervention studies investigating the effect of dietary PUFAs on glycemic regulation in T2D. About half of the studies investigating the effect of fish, fish oils, vegetable oils, or nuts found changes related to glycemic control in people with T2D, while the other half found no effects. Even though some of the studies used SFA as controls, the majority of the included studies compared PUFAs of different quality. Considering that both marine and vegetable oils are high in PUFAs and hence both oils may affect glycemic regulation, the lack of effect in several of the included studies may be explained by the use of an inappropriate control group. It is therefore not possible to draw a firm conclusion, and more studies are needed.

## 1. Introduction

The most important public health challenge in the world today is premature morbidity and mortality from non-communicable diseases (NCDs) like cancer, type 2 diabetes (T2D), and cardiovascular disease (CVD) [1]. Globally, the prevalence of T2D has increased from 108 million in 1980 to 422 million individuals in 2014 [2]. The WHO has estimated that diabetes will be the seventh most important cause of death in the world by 2030 [3]. Relative risk of CVD is increased two to four times in people with T2D compared with non-diabetic subjects, and is the primary cause of death in people with T2D [4].

The health impact of diet is well recognized, and even small dietary changes may contribute to significant health effects [5,6,7]. Lifestyle and diet can be highly effective in preventing and treating T2D [8,9,10,11]. A high intake of saturated fatty acids (SFAs) increases the risk of CVD due to increased low-density lipoprotein (LDL) cholesterol in the blood, while polyunsaturated fatty acids (PUFAs) have the opposite effect [5,12,13,14,15]. Studies indicate that dietary fat quality also may affect insulin sensitivity and hence, the risk of T2D. As early as 1959, Kinsell et al. reported on fat quality and insulin regulation [16]. Several studies on fat quality and glycemic regulation have been published since then [17,18,19,20]. According to observational studies, both intake of PUFAs and replacement of SFAs with PUFAs reduce the risk of T2D [21,22]. Imamura and colleagues recently performed a comprehensive meta-analysis and systematic review of dietary fat and glycemic regulation in randomized controlled trials (RCTs). They found that replacing the intake of SFAs with PUFAs improved glycemia and insulin resistance [19]. These results are in accordance with a systematic review from 2014 [13]. However, none of these reviews distinguished between PUFAs derived from marine or vegetable sources, and both people with and without T2D were included.

In addition to the opposing health effects of saturated versus unsaturated fat, specific PUFAs may differ in their health effects. Some studies have indicated that n-6 PUFAs, but not n-3 PUFAs, may improve insulin sensitivity [13]. In a meta-analysis from 2008, n-3 PUFA supplementation in people with T2D had no significant effect on glycemic control [23], whereas vegetable PUFAs were found to reduce fasting insulin and Homeostasis Assessment Model-Insulin Resistance (HOMA-IR) in a more recent meta-analysis in healthy subjects [24]. To what degree PUFAs from different sources affect glycemic regulation in people with T2D remains unknown.

The aim of the present review was therefore to summarize the literature on human intervention studies investigating the effect of marine- and vegetable-derived PUFAs on glycemic regulation in T2D.

## 2. Materials and Methods

**Figure 1:**
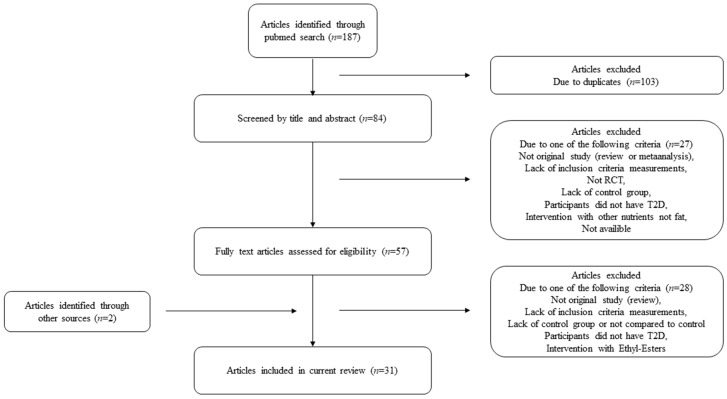
Flow chart of the study selection. RCT: randomized controlled trial; T2D: type 2 diabetes.

To summarize the effects of PUFAs on glycemic control in people with T2D we performed a literature search in PubMed in August 2018. Only original articles on RCTs in humans were included. Furthermore, only studies with information about glycemic control and/or T2D and/or dietary unsaturated fat were included. The search words were: “glycemic control” AND “type 2 diabetes” AND “PUFA” AND/OR “unsaturated fats” AND/OR “nuts” AND/OR “oils” AND/OR “fatty fish” AND/OR “omega 3” AND/OR “omega 6”. We included studies which clearly or possibly fulfilled the following criteria: glycemic control, T2D and dietary interventions, and intake of unsaturated fat. In addition, studies with subjects referred to as non-insulin dependent diabetes mellitus (NIDDM) were also included, and hence these subjects are referred to as NIDDM in the present review. Moreover, we excluded studies that clearly fulfilled at least one of the following criteria: Non original study (for example editorial, review or conference paper), studies that did not compare the criteria measurements to a control group, animal study, articles written in languages other than English, or lack of inclusion criteria measurements (as defined previously). Interventions with ethyl esters and not available articles were excluded, and duplicate articles were removed. In addition to the literature search, two articles were included based on other reviews. In total, 31 articles were identified as eligible and included in the present article. Figure 1 shows the flow chart of the study selection.

## 3. Results and Discussion

In the present review, we identified 31 RCTs (postprandial and short- and long-term intervention trials (lasting from 30 days to 30 weeks), parallel and crossover design) investigating the effects of PUFAs as dietary components in glycemic regulation in people with T2D or NIDDM (Table 1, Table 2 and Table 3). Of the 31 included studies, 14 studies investigated the effect of fish, fish oil, eicosapentaenoic acid (EPA) and/or docosahexaenoic acid (DHA) on glycemic regulation [25,26,27,28,29,30,31,32,33,34,35,36,37,38]; 12 studies investigated vegetable oils [39,40,41,42,43,44,45,46,47,48,49,50]; and five studies investigated the effect of nuts [51,52,53,54,55]. All participants were adults, male and females in the age range between 34 and 84 years (min–max), with T2D or NIDDM.

### 3.1. Fish and Fish Oil

A relationship between fish and/or marine n-3 fatty acids consumption and reduced risk of CVD was originally suggested by epidemiological studies among Greenland Inuits and Danes [56]. The effects of both the amount and quality of dietary fat and fish oil have since been studied intensively. Suggested mechanisms of the cardiovascular benefits from marine n-3 PUFAs include decreased plasma triglyceride levels and blood pressure, as well as anti-thrombotic, antiarrhythmic, and anti-inflammatory effects [57,58,59]. The effect of fish oil on glycemic regulation in T2D is less studied. In the present review, 14 intervention studies with fish or fish oil in people with T2D or NIDDM were included (Table 1).

Balfegó and coworkers investigated the effect of a standard diet with sardines on metabolic control [27]. Thirty-five subjects were randomized to follow either a T2D standard diet (control group), or a T2D standard diet enriched with 100 g of sardines per day, 5 days a week (sardine group) for 6 months. The changes in fasting glucose, glycated hemoglobin A1c (HbA1c), fasting insulin and HOMA-IR values were similar between the two groups [27]. The effects of moderate aerobic exercise and the incorporation of fish into a low-fat diet (30 energy (E) % fat) on glycemic control were examined in 49 subjects [34]. The subjects were randomly assigned to a low fat diet (30 E% fat) with or without one fish meal per day (3.6 g n-3 fatty acids) and further randomized to a moderate (55–65% VO_2_ max) or light (heart rate <100 bpm) exercise program for eight weeks. While fasting insulin levels decreased in the fish and light exercise group compared with light exercise alone (control), there were no differences in fasting blood glucose concentration after any of the interventions compared with the control group. In the fish and light exercise group, they also demonstrated a significant rise in HbA1c compared with the control group [34].

The effects of the marine n-3 fatty acids EPA/DHA in a liquid diet on glycemic control was investigated in a multicenter randomized trial with 30 elderly [29]. The subjects were divided into two groups receiving either an EPA/DHA-rich diet (EPA 25 mg/100 kcal and DHA 17 mg/100 kcal) or a diet without EPA/DHA (control group). A significant reduction in fasting blood glucose and HbA1c was observed after intake of EPA/DHA compared with the control diet [29]. Sarbolouki et al. included 67 men and women in a double blind, placebo-controlled randomized study to investigate the effects of EPA on glycemic regulation [28]. The participants received either EPA (2 g/day, 95% pure EPA) or placebo (2 g corn oil/day) for three months. EPA treatment reduced fasting plasma glucose, HbA1c, and HOMA-IR compared with the control group [28]. In a randomized, double blind, placebo-controlled trial, Wang and colleagues investigated the effect of 4 g fish oil per day (1.34 g EPA and 1.07 g DHA) or corn oil (control) on glucose metabolism in 99 subjects [25]. There were no significant effects on fasting serum glucose, insulin, HbA1c, or HOMA-IR after fish oil treatment for six months compared with corn oil [25]. Zhen et al. investigated the effects of n-3 PUFA from marine or vegetable sources on glycemic control in 166 subjects [26]. The study was a double blind RCT and the participants received either fish oil (2 g/day EPA + DHA), flaxseed oil (2.5 g/day alpha linolenic acid (ALA)), or corn oil (control group) for 180 days. Intake of fish oil, but not flaxseed oil, reduced HbA1c compared with corn oil. There were no effect on fasting insulin or glucose [26]. In order to determine the effects of n-3 fatty acids, a randomized, double blind, placebo-controlled trial was conducted in 81 subjects [31]. The subjects received capsules with either n-3 fatty acids (1.6 g/day EPA and 0.8 g/day DHA) or sunflower oil (control group) for two months. Treatment with n-3 fatty acids significantly decreased HbA1c, while fasting blood glucose was not significantly altered [31]. In line with these results, 6 g/day of either fish oil (1.8 g n-3 PUFAs) or sunflower oil (control) given to 10 men for two months did not show any significant changes in fasting blood glucose, insulin, or HbA1c between the groups [33]. The study had a randomized, double blind crossover design. In the same study, supplementation with fish oil did not alter basal hepatic glucose production and there were no difference in insulin suppression of hepatic glucose production nor in insulin stimulation of whole-body glucose disposal measured by the euglycemic-hyperinsulinemic clamp between the groups [33]. In a double blind RCT, Pedersen and colleagues investigated the impact of vitamin E-enriched fish oil in 44 subjects [32]. The participants received a daily dose of 4 g of either fish oil (2.6 g/day EPA + DHA) or corn oil (control) for eight weeks, in addition to an equal amount of vitamin E (53.6 g/day). There were no significant changes in fasting blood glucose or HbA1c between the groups. However, within the fish oil group, fasting blood glucose was increased [32]. In a double blind, randomized crossover study, 10 g of fish oil (1.8 g EPA + 1.2 g DHA) or olive oil (control) were given daily to eight male subjects for two weeks [37]. There were no significant changes in fasting blood glucose, average daily blood glucose, hyperglycemic clamp, nor insulin sensitivity [37]. In a double blind, randomized, crossover study, 11 subjects received supplements with fish oil, linseed oil, or olive oil (control) for three months in a dose corresponding to 35 mg fatty acids per kilogram body weight [35]. Neither fish oil nor linseed oil modulated glycemic control (fasting glucose and insulin, HbA1c, insulin sensitivity, glucose effectiveness, acute insulin response to glucose) compared with the control group [35]. Morgan and colleagues gave 40 subjects (18 men and 22 women) 9 g of fish oil, 18 g of fish oil, 9 g of corn oil, or 18 g of corn oil daily as a supplement for 12 weeks [36]. They did not detect any effect within (9 g versus 18 g) nor between (fish oil groups combined versus corn oil groups combined) the intervention groups on fasting glucose or HbA1c [36]. In a study by Borkman et al., 10 subjects were included in a three week blinded crossover study [38]. Subjects were given a standard diabetic diet supplemented with a daily dose of 10 g fish oil concentrate (30% n-3 fatty acids), 10 g safflower oil, or no supplementation (control). Fasting blood glucose increased with 14% during fish oil and 11% during safflower oil supplementation compared with control, whereas fasting insulin level remained unchanged [38].

Taken together, about half of the included studies (eight out of 14) found no significant changes related to markers of glycemic regulation such as fasting glucose, insulin, HbA1c or markers related to insulin resistance or sensitivity [25,27,30,32,33,35,36,37] after intervention with fish or fish oil when compared with a control group. In five of the studies a reduction in glucose [28,29], insulin [28,34] and/or HbA1c [26,28,29,31] was observed. Impaired glycemic regulation was observed in two studies, where intake of fish or fish oil increased fasting glucose [38] and an increase in HbA1c [34] was observed after intake of fish in combination with light exercise. In addition to the between groups effects, two of the studies found an additional effect on glycemic regulation within groups, in which Dunstan et al. observed increased HbA1c [34], while Balfego et al. found decreased effect on fasting insulin, HbA1c, and HOMA-IR [27]. In a recent meta-analysis of 11 RCTs including people with T2D, overweight individuals, or healthy individuals, Akinkuolie et al. showed that consumption of n-3 PUFAs did not affect insulin [60]. In line with this, a lack of association between n-3 PUFAs in blood and risk of T2D was the conclusion in the Uppsala Longitudinal Study of Adult Men (ULSAM) [61].

Since about half of the studies in the present review reported improved glycemic regulation while the other half reported no or impaired effects, it is difficult to draw a firm conclusion about fish and fish oil and glycemic regulation.

### 3.2. Vegetable Oils

Vegetable oils are rich in PUFAs, the main constituent being n-6 fatty acids, and in particular linoleic acid (LA). There is convincing evidence that partial replacement of SFAs with monounsaturated fatty acids (MUFAs) or PUFAs lowers fasting blood total- and LDL-cholesterol [5,13,14,15] and thereby reduces the risk of developing CVDs [5,6,13,14,62,63,64]. In addition to the cholesterol-lowering effects of PUFAs, some studies indicate that PUFAs may improve glycemic regulation [13,19]. Twelve studies investigating the effect of vegetable oils on glycemic control in people with T2D or NIDDM were included in this review (Table 2).

Foster and colleagues examined markers of glycemic control in 43 postmenopausal women after intake of flaxseed oil (high in alpha-linolenic acid (ALA)) for 12 weeks [40]. The study was a randomized, double blind, placebo-controlled trial, and the participants received either 40 mg/day zinc, 2 g/day flaxseed oil, both zinc and flaxseed oil, or olive oil (control). There were no significant changes in blood glucose, insulin, HbA1c or HOMA-IR between the intervention groups after 12 weeks. However, insulin and HOMA-IR decreased within the control group [40]. In a study by Jenkins et al., the combined effect of ALA, MUFAs, and low glycemic load on glycemic control and CVD risk factors were investigated in 141 subjects [41]. The study was a RCT with a parallel design and the subjects were provided daily with canola oil-enriched whole-wheat bread (500 kcal/day or 31 g canola oil per 2000 kcal) or whole-wheat bread without canola oil (500 kcal) (control diet). The test diet significantly reduced HbA1c compared with the control diet and the result remained statistically significant after adjustment for body weight change [41]. Barre and coworkers investigated the effect of flaxseed oil on glycemic control in 32 subjects [44]. The subjects were randomly assigned to receive 10 g/day of flaxseed oil or safflower oil for three months. The amount of ALA was approximately 60 mg/kg body weight/day in the flaxseed oil group. Flaxseed oil had no impact on fasting blood glucose, insulin, or HbA1c compared with the control group [44]. Conjugated linoleic acid (CLA), the *trans* fatty acid of LA, was compared with safflower oil high in *cis*-LA in 35 obese, postmenopausal women [43]. The participants consumed 8 g oil per day for 16 weeks in a crossover study, with four weeks washout in between the intervention periods, giving a total of 36 weeks. The aim of the study was to investigate weight reduction, and they found a significant reduction in BMI after CLA oil intake but not after safflower oil intake. Nevertheless, even though a weight reduction is associated with improved glycemic regulation, there was a significant reduction in fasting blood glucose only within the safflower oil group [43]. To investigate the long-term effects of a diet enriched in LA on insulin sensitivity, Heine and colleagues conducted a randomized, crossover study in 14 subjects [50]. The PUFA to SFA ratio (P:S ratio) of the diets were altered by substituting LA-rich fats and oils for products rich in saturated fats. The participants received a diet (total fat content of 38–39 E%) with a P:S ratio of 0.3 (low P:S diet) or 1.0 (high P:S diet) in a randomized order for 30 weeks each. Fasting blood glucose, insulin, HbA1c, glucose incremental area under the curve (iAUC), C-peptide, and insulin responses did not differ between the groups after intervention. However, the metabolic clearance rate of glucose was higher in the high P:S diet compared with the control group. This difference was only observed at the lowest infusion rate (6 mg/kg/min) [50]. A 12-week intervention with bakery products containing flaxseed oil (13 g/day), milled flaxseed (32 g/day), or no flaxseed (control group) investigated the effects on fasting blood glucose, insulin, and HbA1c in 34 adult males and females [42]. The flaxseed and flaxseed oil groups received equivalent amounts of 7.4 g ALA per day. There were no differences in fasting HbA1c, glucose and insulin after the intervention period [42]. Isocaloric diets with different fatty acid composition was investigated in a randomized crossover trial with 16 obese subjects for six weeks [48]. The energy content of carbohydrate, protein and fats were kept constant, but the diets differed in fat composition. There were no significant changes between the diets in HbA1c, fasting blood glucose, insulin, or postprandial glycemic response. However, serum insulin and C-peptide responses increased following the *trans*-MUFA and SAT diets compared with the *cis*-MUFA diet [48]. In a study by Gerhard et al., the effect of two ad libitum diets on glycemic control was investigated [45]. Eleven subjects were randomly assigned to receive an ad libitum low-fat, high-carbohydrate diet (20 E% total fat, 65 E% carbohydrates, higher in fiber), or a high-MUFA diet (40 E% total fat, 26 E% MUFAs, 45 E% carbohydrates), each for six weeks. There were no effect on fasting glucose or HbA1c after the high-MUFA diet compared with low-fat diet [45]. Brynes and coworkers investigated the effect of an isoenergetic high-MUFA diet (olive oil) compared with high-PUFA diet (corn oil), on glycemic regulation in nine overweight subjects [46]. Glycemic control remained stable throughout the study and there were no change in fasting or postprandial iAUC for glucose or insulin in response to an identical standard meal after 24 days of intervention [46]. Instead of comparing fat quality, intake of MUFAs from oil or margarine was compared with intake of carbohydrates from breakfast cereals with either a high or low glycemic index [47]. After a six-month intervention with 72 subjects there were no differences in fasting blood glucose or HbA1c between the groups. After a standard breakfast and lunch, a reduction in mean 8-h plasma insulin in the group given MUFAs compared with the cereal group was however observed [47]. During a four-week period, 12 women received an isocaloric diet high in either MUFAs (HMUFA) or complex carbohydrates (high-CHO). This crossover study had a four-week washout period during which the subjects followed the American Diabetes Association (ADA) isocaloric diet. Glycemic control, including fasting blood glucose, insulin and fructosamine did not significantly change with the different intervention diets. However, fasting blood glucose and insulin were reduced within both groups after intervention [49]. The effect of walnut oil on blood glucose in 90 subjects was investigated in a RCT, lasting for three months [39]. In the experimental group, walnut oil (15 g/day) was added to the diet, while the control group did not undergo any intervention. HbA1c level and fasting blood glucose decreased significantly in the experiment group compared with the control group [39].

In summary, six of the 12 studies investigating vegetable oils on glycemic regulation such as fasting glucose, insulin, HbA1c or markers related to insulin resistance or insulin sensitivity did not find any effects in people with T2D [40,42,44,45,46,49], although two of the studies found within group changes [40,49]. In the other six studies however, there were changes in glycemic regulation either between or within groups [39,41,43,47,49,50]. In contrast to the studies showing a decreasing effect on glycemic regulation after intervention, the study by Norris et al. found increased fasting glucose levels and HOMA-IR after intervention with CLA compared with safflower oil (control group). However, safflower oil reduced both fasting glucose levels and HOMA-IR within the control group [43]. Even though CLA, a *trans* fatty acid, is debated for its possible health effects [65], *trans* fatty acids in general are well known for their cholesterol increasing effects [66] and may explain the impaired effects related to glycemic regulation. Vegetable oils mainly consist of n-6 PUFAs and in particular LA, and other studies have shown a beneficial effect of n-6 PUFAs on glycemic regulation. A recent pooled analysis from prospective cohort studies demonstrated that higher levels of LA in blood were associated with a 43% reduced relative risk for T2D [67]. This is in line with the results from the ULSAM study. Men who developed T2D had a lower proportion of LA and a higher proportion of SFAs (C:14 and C:16) in serum cholesterol esters compared with those who did not develop T2D [61]. Summers et al. showed that switching from a diet rich in SFAs to a diet rich in PUFAs for 5 weeks improved insulin sensitivity in people with T2D, non-obese and obese subjects [68]. Even though others have found improved glycemic regulation after intervention with PUFAs, we are not able to draw firm conclusions based on the studies included in the present review.

### 3.3. Nuts

Nuts are high-energy, nutrient-dense foods that are rich in PUFAs and other bioactive components, including fiber, antioxidants, vitamins and minerals [69]. Epidemiological studies have found an inverse relationship between nut consumption and reduced risk of T2D [70,71]. In the present review, five RCTs intervening with different nuts (cashew, pistachio, peanuts or mixed nuts) in people with T2D were included (Table 3).

In the study by Mohan and coworkers, they investigated the effect of a standard diabetic diet with 30 g cashew nuts per day for 12 weeks on glycemic regulation in 300 subjects [51]. They did not find any significant differences in glycemic regulation (fasting blood glucose, insulin, HbA1c, and HOMA-IR) after the intervention [51]. In another study, by Parham et al., the effect of pistachio nut supplementation on glycemic control and inflammatory markers was investigated [53]. The study included 48 subjects in a double blind, randomized, placebo-controlled crossover trial. The subjects received either 25 g pistachio nuts twice a day or a control diet without nuts for 12 weeks, followed by an eight-week washout period, before switching interventions. A decrease in HbA1c and fasting blood glucose was observed after intake of pistachio nuts compared with the control group. There were no effects on HOMA-IR after intake of pistachio nuts [53]. Also, Sauder and coworkers investigated the effect of pistachio nuts on glycemic control [52]. They included 30 subjects in a randomized, controlled, crossover study. After a two-week run-in period, participants consumed diets with pistachio nuts (contributing with 20% of total energy) or without pistachio nuts (control group) for four weeks each, separated by a two-week washout period. Glycemic measures were assessed both fasted and during a 75-g oral glucose tolerance test. There were no effect on fasting glucose, insulin, HbA1c, HOMA-IR or glucose area under the curve (AUC) or insulin AUC after intake of nuts compared with control group [52]. Wien and colleagues investigated the effect of incorporating peanuts into the American Diabetes Association (ADA) meal plan on cardio-metabolic parameters [54]. They performed a 24-week parallel RCT with 60 subjects. The intervention group received an ADA meal plan containing about 20% of energy from peanuts, while the control group followed a peanut-free ADA meal plan. After 24 weeks of intervention, there were no differences in fasting blood glucose or HbA1c between the groups. [54]. In a study by Kendall and colleagues, the effect of nut consumption alone or in combination with white bread on postprandial glycaemia in 14 healthy compared with 10 people with T2D were examined [55]. The participants consumed 30, 60, and 90 g of mixed nuts alone or in combination with white bread (50 g available carbohydrate). All three doses of mixed nuts consumed alone significantly reduced the glycemic response compared with the control group. Adding nuts (60 g and 90 g) to white bread significantly reduced the glycemic response in healthy subjects however, significant reduction in glycemic response were only observed after adding 90 g nuts to white bread in people with T2D [55].

Taken together, three of the five studies investigating intake of nuts and glycemic regulation such as fasting blood glucose, insulin, HbA1c or markers related to insulin resistance or sensitivity found beneficial effects in T2D. Pistachio nuts reduced both fasting blood glucose levels and HbA1c [53] or fructosamine [52], and intervention with mixed nuts led to reduction in postprandial glycemic response [55]. In these studies, nut consumption benefits glycemic regulation regardless of the type of nuts, study design or duration. These results are in line with the The Prevención con Dieta Mediterránea (PREDIMED)-study, in which 30 g nuts per day (almonds, hazelnuts, and walnuts), given as supplements to a Mediterranean diet, significantly reduced the incidence of T2D compared with a low-fat diet without nut supplementation in high risk subjects [72].

## 4. Discussion

In the present summary, improvements related to glycemic control in people with T2D were observed in about half of the studies investigating the effect of fish, fish oil, or vegetable oil. Intake of nuts may however indicate a more beneficial effect, even though the number of studies are limited. The present review also demonstrates that the studies investigating the effect of PUFAs on glycemic control in subjects with T2D or NIDDM are quite different in design with respect to type of dietary intervention, study duration, and measurements of glycemic control, and hence the results are difficult to compare. Most importantly, the intervention and the control food differ largely between the studies. Of the included studies, mainly vegetable oils (corn, sunflower, linseed, and olives) functioned as control for both fish and fish oil interventions, and for different vegetable oils. Hence, the studies are comparing PUFAs of different quality. Considering that vegetable oils are high in PUFAs and therefore may affect glycemic regulation, the lack of effect in several of the included studies may be explained by the use of an inappropriate control group. It is therefore not possible to conclude whether intake of marine- or vegetable-derived PUFAs will have a positive effect on glycemic regulation in people with T2D. In the previous mentioned meta-analysis performed by Imamura et al., intake of PUFAs was compared with intake of SFAs. Changing the intake of SFAs with PUFAs improved glycaemia and insulin resistance [19]. SFAs may therefore represent a better control group when investigating the effect of PUFAs on glycemic regulation. The study by Imamura et al. was however not unique to T2D, as both healthy and people with T2D were included. This may explain the discrepant findings between previous studies and the present review. In addition, Coelho et al. conclude that supplementation of 0.42–5.2 g PUFAs per day for at least eight weeks may become an alternative treatment for T2D. However, only six studies were included in the review [20]. In contrast, a meta-analysis from 2011 did not find any effect of n-3 PUFA consumption on insulin sensitivity. The study included 11 studies investigating the effect in both healthy and people with T2D [60]. In addition, ALA-enriched diets did not affect HbA1c, fasting blood glucose, or insulin in a meta-analysis conducted in people with T2D. The study included eight interventions [73]. In conclusion, the reported discrepancies between other studies and this review regarding PUFAs and glycemic control are probably due to the heterogeneity of the studies.

Even though fat quality has been shown to affect glycemic regulation, it is possible that also fat quantity will be of importance. Vessby and coworkers reported that a total fat intake of more than 37 E% increases the risk of insulin resistance independent of fat quality [74]. Total fat intake were not consistently reported in the present reviewed studies, and hence we cannot rule out that a high total fat intake may have affected the results.

Limitations of the current review includes the search strategy. To ensure that the included studies had focus on glycemic regulation, the search words “glycemic control” were used. This may have affected the number of articles and we cannot rule out the possibility that some relevant studies have not been included. We did however include two studies from other reviews.

## 5. Conclusions

In the present review, we have identified studies that show beneficial effects of both marine and vegetable-derived PUFAs on glycemic control in people with T2D. The studies are however different in design and no firm conclusions can be drawn. In order to understand the role of PUFAs in the management of T2D, we suggest more well designed RCTs where the effect of PUFAs specifically is compared with the effect of SFAs.

## Figures and Tables

**Table 1:** Effects of fish and fish oil on glycemic regulation in type 2 diabetes in randomized controlled trials. Significant results are indicated by an up/down arrow.

Study	Subject Characteristics	Study Design	Intervention	Glucose	Insulin	HbA1c	Other Markers
Wang et al. 2017, European Journal of Nutrition, China	*n* = 99, T2D, 65 years, M/F	6 months Parallel	(1) Corn oil (4 g/day) (2) Fish oil (4 g/day (1.34 g EPA and 1.07 g DHA))	↔	↔	↔	↔ HOMA-IR
Zheng et al. 2016, Mol. Nutr. Food Res, China	*n* = 166, T2D, 59 years, M/F	180 days Parallel	(1) Corn oil (1 g/day) (2) Fish oil (1 g/day (500 mg EPA + DHA, EPA:DHA = 3:2)) (3) Flaxseed oil (1 g/day (630 mg ALA))	↔	↔	(2) ↓ (3) ↔	↔ HOMA-IR
Balfegó et al. 2016, Lipids Health Dis, Spain	*n* = 35, T2D, 60 years, M/F	6 months Parallel	(1) Standard diet (2) Standard diet enriched with sardines 5 days a week (100 g/day)	↔	↔ *Within groups:* (1), (2) ↓	↔ *Within groups:* (1) ↓	↔ HOMA-IR *Within groups:* (1), (2) ↓ HOMA-IR
Sarbolouki et al. 2013, Singapore Med J, Iran	*n* = 67, T2D, 45 years, M/F	3 months Parallel	(1) Corn oil (2 g/day) (2) EPA (2 g/day)	↓	↓	↓	↓ HOMA-IR
Ogawa et al. 2013, Tohoku J Exp Med, Japan	*n* = 30, T2D, 80 years, M/F	3 months Parallel	(1) Liquid diet without EPA/DHA (2) Liquid diet containing EPA (25 mg/100 kcal) and DHA (17 mg/100 kcal)	↓		↓	
Crochemore et al. 2012, Nutr Clin Pract, Brazil	*n* = 41, T2D, 61 years, F	30 days Parallel	(1) Gelatin (500 mg/capsule) (2) Fish oil (2.5 g/day (547.5 mg EPA + 352.5 mg DHA)) (3) Fish oil (1.5 g/day (328.5 mg EPA + 211.5 mg DHA))	↔	↔	↔	↔ HOMA-IR ↔ QUICKI
Pooya et al. 2010, Nutrition, Metabolism & Cardiovascular Disease, Iran	*n* = 81, T2D, Control: 53 years Treatment: 56 years M/F	2 months Parallel	(1) Sunflower oil (2100 mg/day (12% SFA, 71% linoleic acid (LA), 16% MUFA)) (2) n-3 (3 g/day (1584 mg/day EPA, 828 mg/day DHA, 338 mg other n-3))	↔		↓	
Pedersen et al. 2003, EJCN, Denmark	*n* = 44, T2D, 63 years, M/F	8 weeks Parallel	(1) Corn oil (4 g/day) (2) Fish oil (4 g/day (2.6 g EPA + DHA))	↔ *Within groups:* (2) ↑		↔	
Luo et al. 1998, Diabetes Care, France	*n* = 10, T2D, 54 years, M	2 × 2 months Crossover	(1) Sunflower oil (6 g/day(65% n-6, 0,2% n-3, 24% MUFA, 11% SFA)) (2) Fish oil (6 g/day (1.8 g n-3: 18% EPA, 12% DHA, 4% n-6, 36% MUFA, 30% SFA))	↔	↔	↔	↔ euglycemic-hyperinsulinemic clamp
Dunstan et al. 1997, Diabetes Care, Australia	*n* = 49, NIDDM, 52–54 years, M/F	8 weeks parallel	(1) Light exercise (2) Fish (3.6 g n-3/day) and light exercise (3) Fish (3.6 g n-3/day) and moderate exercise (4) Moderate exercise	↔	(2) ↓ (3) (4) ↔	(2) ↑ (3) (4) ↔	
McManus et al. 1996, Diabetes Care, Canada	*n* = 11, T2D, 62 years, M/F	3 × 3 months Crossover	(1) Linseed oil (35 mg FA/kg body weight/day) (2) Fish oil (35 mg FA/kg body weight/day)	↔	↔	↔	↔ Insulin sensitivity ↔ Glucose effectiveness ↔ Acute insulin response to glucose
Morgan et al. 1995, Diabetes Care, USA	*n* = 40, NIDDM, 54 years, M/F	12 weeks Parallel	(1) Corn oil (9 g/day) (2) Corn oil (18 g/day) (3) Fish oil (9 g/day) (4) Fish oil (18 g/day)	↔		↔	
Annuzzi et al. 1991, Atherosclerosis, Italy	*n* = 8, NIDDM, 51 years, M	2 × 2 weeks Crossover	(1) Olive oil (10 g) (2) Fish oil (10 g (1.8 g EPA + 1.2 g DHA))	↔ ↔ daily average	↔ insulin sensitivity		↔ hyperglycemic clamp
Borkman et al. 1989 Diabetes, Austrailia	*n* = 10, NIDDM, 57 years, M/F	3 × 3 weeks Crossover	(1) Standard diabetic diet (2) Standard diabetic diet + Safflower oil (10 g) (3) Standard diabetic diet + Fish oil (10 g)	(2) (3) ↑	↔		↔ hyperinsulinemic-euglycemic clamp

Significant differences (*p* ≤0.05) between intervention group(s) and control group are shown with ↑ or ↓, while ↔ indicates no significant difference. When several intervention groups are present, the results for each group are indicated with number. Fasting values are shown, if not otherwise stated. Control group is referred to as (1). DHA: docosahexaenoic acid; EPA: eicosapentaenoic acid; F: female; g: gram; Gr: group; HbA1c: glycated hemoglobin A1c; HOMA-IR: Homeostasis Assessment Model-Insulin Resistance; M: male; mg: milligram; MUFA: monounsaturated fatty acid; *n*: number; n-3: omega-3; NIDDM: non-insulin dependent diabetes mellitus; PUFA: polyunsaturated fatty acid; SFA: saturated fatty acid; T2D: type 2 diabetes; wt: weight.

**Table 2:** Effects of vegetable oils on glycemic regulation in type 2 diabetes in randomized controlled trials. Significant results are indicated by an up/down arrow.

Study	Subject Characteristics	Study Design	Intervention	Glucose	Insulin	HbA1c	Other Markers
Zibaeenezhad et al. 2016, Int J Endocrin Metab, Iran	*n* = 90, T2D, 55 years, M/F	3 months Parallel	(1) No oil (2) Walnut oil (15 g/day)	↓		↓	
Foster et al. 2013, Journal of Diabetes Research and Clinical Metabolism, Australia	*n* = 43, T2D, 65 years, F	12 weeks Parallel	(1) Olive oil (2000 mg/day + 40 mg/day zinc) (2) Zink (40 mg/d) (3) Flaxseed oil (2000 mg/day (1200 mg ALA)) (4) Zinc and flaxseed oil (40 mg/day zinc + 2000 mg/day flaxseed oil)	↔	↔ *Within groups:* (1) ↓	↔	↔ HOMA-IR *Within groups:* (1) ↓ HOMA-IR
Jenkins et al. 2014, Diabetes Care, Canada	*n* = 141, T2D, 59 years, M/F	3 months Parallel	(1) whole-wheat bread without canola oil (500 kcal/day) (2) low-GL diet with a canola oil-enriched bread (500 kcal/day)	↔		↓	
Taylor et al. 2010, AJCN, Canada	*n* = 34, T2D, 52 years, M/F	12 weeks Parallel	(1) Bakery products with no flaxseed (2) Bakery products with milled flaxseed (32 g/day) (3) Bakery products with flaxseed oil (13 g/day)	↔	↔	↔	↔ HOMA-IR ↔ QUICKI
Norris et al. 2009, AJCN, United States	*n* = 35, T2D, 60 years, F	2 × 16 weeks crossover	(1) Safflower oil (8 g/day) (2) CLA (*c*9*t*11 and *t*10*c*12) (8 g/day)	↑ *Within groups:* (1) ↓	↔		↑ HOMA-IR *Within groups:* (1) ↓ HOMA-IR
Barre et al. 2008, J Ole Sci, Canada	*n* = 32, T2D, 60 years, M/F	3 months Parallel	(1) Safflower oil 10 g/day (control) (2) Flaxseed oil 10 g/day (60 mg ALA/kg body weight/day)	↔	↔	↔	
Gerhard 2004, AJCN, United States	*n* = 11, T2D, 50 years, M/F	6 weeks Parallel	(1) Low-fat diet (total fat 20 E%, carbohydrates 65 E%, higher in fiber) (2) High MUFA diet (total fat 40 E%, MUFA 26 E%, carbohydrates 45 E%)	↔		↔	↔ Fructosamin
Brynes et al. 2000, AJCN, United Kingdom	*n* = 9, T2D, 56 years, M/F	2 × 3 weeks Crossover	(1) high-MUFA isoenergetic diet (olive oil) (2) high-PUFA isoenergetic diet (corn oil)	↔ ↔ iAUC	↔ ↔ iAUC	↔	↔ short insulin tolerance test (SITT)
Tsihlias et al. 2000, AJCN, Canada	*n* = 72, T2D, 42–79 years, M/F	6 months Parallel	(1) High-GI diet (cereals) (10% of energy) (2) Low-GI diet (cereals) (10% of energy) (3) High MUFA diet (margarine and olive oil) (10% of energy)	↔	(3) ↓ mean 8-h insulin (2) ↔ mean 8h insulin	↔	
Christiansen et al. 1997, Diabetes Care, Denmark	*n* = 16, NIDDM, 55 years, M/F	3 × 6 weeks Crossover	(1) SAT diet (20 E% SFA, 5 E% PUFA, 5 E% MUFA) (2) *Cis*-MUFA diet (20 E% *cis*-MUFA, 5 E% PUFA, 5 E% SFA) (3) *Trans*-MUFA diet (20 E% *trans*-MUFA, 5 E% PUFA, 5 E% SFA)	↔ ↔ AUC	↔ (2) ↓ iAUC	↔	(2) ↓ C-peptide iAUC
Lerman-Garber et al., 1994, Diabetes Care, Mexico	*n* = 12, NIDDM, 56 years, F	2 × 4 weeks Crossover	(1) Diet high in MUFA (HMUFA) (olive oil) (2) Diet high in complex carbohydrates (high-CHO)	↔ *Within groups:* (1), (2) ↓	↔ *Within groups:* (1), (2) ↓		↔ Fructosamine
Heine et al. 1989, AJCN, USA	*n* = 14, NIDDM, 52 years, M/F	2 × 30 weeks Crossover	(1) Low P:S diet (P:S ratio 0.3) (2) High P:S diet (P:S ratio 1.0)	↔ ↔ iAUC	↔	↔	↑ Metabolic clearance rate of glucose ↔ C-peptide

Significant differences (*p* ≤0.05) between intervention group(s) and control group are shown with ↑ or ↓, while ↔ indicates no significant difference. When several intervention groups are present, the results are for each group are indicated with number. Fasting values are shown, if not otherwise stated. Control group is referred to as (1). ALA: alpha-linolenic Acid; CHO: carbohydrates; E%: energy %; F: female; g: gram; GI: glycemic index; GL: glycemic load; HbA1c: glycated hemoglobin A1c; HOMA-%β: HOMA–percentage beta cell function; HOMA-IR: Homeostasis Assessment Model-Insulin Resistance; iAUC: incremental area under the curve; M: male; MUFA: monounsaturated fatty acid; *n*: number; NIDDM: non-insulin dependent diabetes mellitus; P:S: PUFA/SFA ratio; PUFA: polyunsaturated fatty acid; SAT: high saturated fatty acid diet; SFA: saturated fatty acid; T2D: type 2 diabetes; QUICKI: quantitative insulin check.

**Table 3:** Effects of nuts on glycemic regulation in type 2 diabetes in randomized controlled trials. Significant results are indicated by an up/down arrow.

Study	Subject Characteristics	Study Design	Intervention	Glucose	Insulin	HbA1c	Other Markers
Mohan et al. 2018, J Nutr, India	*n* = 269, T2D, 51 years, M/F	12 weeks Parallel	(1) Standard diabetic diet (2) Standard diabetic diet plus cashew nuts (30 g/day)	↔	↔	↔	↔ HOMA-IR
Sauder et al. 2015, Metabolism, USA	*n* = 30, T2D, 40–74 years, M/F	2 × 4 weeks Crossover	(1) Control diet; based on the American Heart Association’s Therapeutic Lifestyle Changes diet (26.9% total fat, 6.7% saturated fat, 186 mg/day cholesterol) (2) Pistachios added to the control diet (20 % of daily energy)	↔	↔	↔	↔ HOMA-IR ↔ Matsuda ↓ Fructosamine
Parham et al. 2014, Rev Diabet Study, Iran	*n* = 48, T2D, 50–53 years, M/F	2 × 12 weeks Crossover	(1) Diet without nuts (2) Pistachio nuts (50 g/day)	↓		↓	
Wien et al. 2014, Nutr Journal, USA	*n* = 60, T2D, 34–84 years, M/F	24 weeks Parallel	(1) ADA meal plan without nuts (2) ADA meal plan with peanuts (20% of total energy (mean 46 g/day))	↔		↔	
Kendall et al. 2011, Nutrition, Metabolism & Cardiovascular Diseases, Canada	*n* = 24 T2D: 68 years Healthy: 36 years M/F	2-h Postprandial Crossover	(1) White bread (50 g available carbohydrate) (2) Mixed nuts (30 g) (3) Mixed nuts (60 g) (4) Mixed nuts (90 g) (5) White bread + mixed nuts (30 g) (6) White bread + mixed nuts (60 g) (7) White bread + mixed nuts (90 g)	(2), (3), (4) ↓ iAUC (healthy and T2D) (6), (7) ↓ iAUC (healthy) (7) ↓ iAUC (T2D)			

Significant differences (*p* ≤0.05) between intervention group(s) and control group are shown with ↑ or ↓, while ↔ indicates no significant difference. When several intervention groups are present, the results are for each group are indicated with number. Control group is referred to as (1). ADA: American Diabetes Association; d: day; F: female; g: gram; h: hour; HbA1c: glycated hemoglobin A1c; HOMA-IR: Homeostasis Assessment Model-Insulin Resistance; iAUC: incremental area under the curve; M: male; *n*: number; T2D: type 2 diabetes.

## References

[1] World Health Organization (2018). The Top 10 Causes of Death. https://www.who.int/news-room/fact-sheets/detail/the-top-10-causes-of-death.

[2] World Health Organization (2016). Global Report on Diabetes.

[3] World Health Organization (2018). Diabetes. https://www.who.int/en/news-room/fact-sheets/detail/diabetes.

[4] Yoo J.Y., Kim S.S. (2016). Probiotics and Prebiotics: Present Status and Future Perspectives on Metabolic Disorders. Nutrients.

[5] Ulven S.M., Leder L., Elind E., Ottestad I., Christensen J.J., Telle-Hansen V.H., Skjetne A.J., Raael E., Sheikh N.A., Holck M. (2016). Exchanging a few commercial, regularly consumed food items with improved fat quality reduces total cholesterol and LDL-cholesterol: A double-blind, randomised controlled trial. Br. J. Nutr..

[6] Estruch R., Ros E., Salas-Salvadó J., Covas M.I., Corella D., Arós F., Gómez-Gracia E., Ruiz-Gutiérrez V., Fiol M., Lapetra J. (2018). Primary Prevention of Cardiovascular Disease with a Mediterranean Diet Supplemented with Extra-Virgin Olive Oil or Nuts. N. Engl. J. Med..

[7] GBD 2015 Risk Factors Collaborators (2016). Global, regional, and national comparative risk assessment of 79 behavioural, environmental and occupational, and metabolic risks or clusters of risks, 1990–2015: A systematic analysis for the Global Burden of Disease Study 2015. Lancet.

[8] Knowler W.C., Barrett-Connor E., Fowler S.E., Hamman R.F., Lachin J.M., Walker E.A., Nathan D.M. (2002). Reduction in the incidence of type 2 diabetes with lifestyle intervention or metformin. N. Engl. J. Med..

[9] Lim E.L., Hollingsworth K.G., Aribisala B.S., Chen M.J., Mathers J.C., Taylor R. (2011). Reversal of type 2 diabetes: Normalisation of beta cell function in association with decreased pancreas and liver triacylglycerol. Diabetologia.

[10] Barnard N.D., Katcher H.I., Jenkins D.J., Cohen J., Turner-McGrievy G. (2009). Vegetarian and vegan diets in type 2 diabetes management. Nutr. Rev..

[11] Barnard R.J., Jung T., Inkeles S.B. (1994). Diet and exercise in the treatment of NIDDM. The need for early emphasis. Diabetes Care.

[12] O’Flaherty M., Flores-Mateo G., Nnoaham K., Lloyd-Williams F., Capewell S. (2012). Potential cardiovascular mortality reductions with stricter food policies in the United Kingdom of Great Britain and Northern Ireland. Bull. World Health Organ..

[13] Schwab U., Lauritzen L., Tholstrup T., Haldorsson T.I., Risérus U., Uusitupa M., Becker W. (2014). Effect of the amount and type of dietary fat on cardiometabolic risk factors and risk of developing type 2 diabetes, cardiovascular diseases, and cancer: A systematic review. Food Nutr. Res..

[14] Mozaffarian D., Micha R., Wallace S. (2010). Effects on Coronary Heart Disease of Increasing Polyunsaturated Fat in Place of Saturated Fat: A Systematic Review and Meta-Analysis of Randomized Controlled Trials. PLoS Med..

[15] Jakobsen M.U., O’Reilly E.J., Heitmann B.L., Pereira M.A., Bälter K., Fraser G.E., Goldbourt U., Hallmans G., Knekt P., Liu S. (2009). Major types of dietary fat and risk of coronary heart disease: A pooled analysis of 11 cohort studies123. Am. J. Clin. Nutr..

[16] Kinsell L.W., Michaels G.D., Olson F.E., Coelho M., McBride Y., Fukayama G., Conklin J., Walker G. (1959). Dietary Fats and the Diabetic Patient. N. Engl. J. Med..

[17] Mann J. (2006). Nutrition Recommendations for the Treatment and Prevention of Type 2 Diabetes and the Metabolic Syndrome: An Evidenced-Based Review. Nutr. Rev..

[18] Riserus U., Willett W.C., Hu F.B. (2009). Dietary fats and prevention of type 2 diabetes. Prog. Lipid Res..

[19] Imamura F., Micha R., Wu J.H.Y., Otto M.C.D.O., Otite F.O., Abioye A.I., Mozaffarian D. (2016). Effects of Saturated Fat, Polyunsaturated Fat, Monounsaturated Fat, and Carbohydrate on Glucose-Insulin Homeostasis: A Systematic Review and Meta-analysis of Randomised Controlled Feeding Trials. PLoS Med..

[20] Coelho O.G.L., da Silva B.P., Rocha D.M.U.P., Lopes L.L., Alfenas R.C.G. (2017). Polyunsaturated fatty acids and type 2 diabetes: Impact on the glycemic control mechanism. Crit. Rev. Food Sci. Nutr..

[21] Meyer K.A., Kushi L.H., Jacobs D.R., Folsom A.R. (2001). Dietary Fat and Incidence of Type 2 Diabetes in Older Iowa Women. Diabetes Care.

[22] Salmerón J., Hu F.B., Manson J.E., Stampfer M.J., Colditz G.A., Rimm E.B., Willett W.C. (2001). Dietary fat intake and risk of type 2 diabetes in women. Am. J. Clin. Nutr..

[23] Hartweg J., Perera R., Montori V.M., Dinneen S.F., Neil A.H., Farmer A.J. (2008). Omega-3 polyunsaturated fatty acids (PUFA) for type 2 diabetes mellitus. Cochrane Database Syst. Rev..

[24] Wanders A.J., Blom W.A.M., Zock P.L., Geleijnse J.M., Brouwer I.A., Alssema M. (2019). Plant-derived polyunsaturated fatty acids and markers of glucose metabolism and insulin resistance: A meta-analysis of randomized controlled feeding trials. BMJ Open Diabetes Res. Care.

[25] Wang F., Wang Y., Zhu Y., Liu X., Xia H., Yang X., Sun G. (2017). Treatment for 6 months with fish oil-derived n-3 polyunsaturated fatty acids has neutral effects on glycemic control but improves dyslipidemia in type 2 diabetic patients with abdominal obesity: A randomized, double-blind, placebo-controlled trial. Eur. J. Nutr..

[26] Zheng J.S., Lin M., Fang L., Yu Y., Yuan L., Jin Y., Feng J., Wang L., Yang H., Chen W. (2016). Effects of n-3 fatty acid supplements on glycemic traits in Chinese type 2 diabetic patients: A double-blind randomized controlled trial. Mol. Nutr. Food Res..

[27] Balfegó M., Canivell S., Hanzu F.A., Sala-Vila A., Martínez-Medina M., Murillo S., Mur T., Ruano E.G., Linares F., Porras N. (2016). Effects of sardine-enriched diet on metabolic control, inflammation and gut microbiota in drug-naïve patients with type 2 diabetes: A pilot randomized trial. Lipids Heal..

[28] Sarbolouki S., Javanbakht M., Derakhshanian H., Hosseinzadeh P., Zareei M., Hashemi S., Dorosty A., Eshraghian M., Djalali M. (2013). Eicosapentaenoic acid improves insulin sensitivity and blood sugar in overweight type 2 diabetes mellitus patients: A double-blind randomised clinical trial. Singap. Med. J..

[29] Ogawa S., Abe T., Nako K., Okamura M., Senda M., Sakamoto T., Ito S. (2013). Eicosapentaenoic Acid Improves Glycemic Control in Elderly Bedridden Patients with Type 2 Diabetes. Tohoku J. Exp. Med..

[30] Crochemore I.C.C., Souza A.F., de Souza A.C., Rosado E.L. (2012). Omega-3 polyunsaturated fatty acid supplementation does not influence body composition, insulin resistance, and lipemia in women with type 2 diabetes and obesity. Nutr. Clin. Pract..

[31] Pooya S., Jalali M.D., Jazayery A.D., Saedisomeolia A., Eshraghian M.R., Toorang F. (2010). The efficacy of omega-3 fatty acid supplementation on plasma homocysteine and malondialdehyde levels of type 2 diabetic patients. Nutr. Metab. Cardiovasc. Dis..

[32] Pedersen H., Petersen M., Major-Pedersen A., Jensen T., Nielsen N.S., Lauridsen S.T., Marckmann P. (2003). Influence of fish oil supplementation on in vivo and in vitro oxidation resistance of low-density lipoprotein in type 2 diabetes. Eur. J. Clin. Nutr..

[33] Luo J., Rizkalla S.W., Vidal H., Oppert J.-M., Colas C., Boussairi A., Guerre-Millo M., Chapuis A.-S., Chevalier A., Durand G. (1998). Moderate Intake of n-3 Fatty Acids for 2 Months Has No Detrimental Effect on Glucose Metabolism and Could Ameliorate the Lipid Profile in Type 2 Diabetic Men: Results of a controlled study. Diabetes Care.

[34] Dunstan D.W., Mori T.A., Puddey I.B., Beilin L.J., Burke V., Morton A.R., Stanton K.G. (1997). The Independent and Combined Effects of Aerobic Exercise and Dietary Fish Intake on Serum Lipids and Glycemic Control in NIDDM: A randomized controlled study. Diabetes Care.

[35] McManus R.M., Jumpson J., Finegood D.T., Clandinin M.T., Ryan E.A. (1996). A Comparison of the Effects of n-3 Fatty Acids from Linseed Oil and Fish Oil in Well-Controlled Type II Diabetes. Diabetes Care.

[36] Morgan W.A., Raskin P., Rosenstock J. (1995). A Comparison of Fish Oil or Corn Oil Supplements in Hyperlipidemic Subjects with NIDDM. Diabetes Care.

[37] Annuzzi G., Rivellese A., Capaldo B., Di Marino L., Iovine C., Marotta G., Riccardi G. (1991). A controlled study on the effects of n − 3 fatty acids on lipid and glucose metabolism in non-insulin-dependent diabetic patients. Atherosclerosis.

[38] Borkman M., Chisholm D.J., Furler S.M., Storlien L.H., Kraegen E.W., Simons L.A., Chesterman C.N. (1989). Effects of Fish Oil Supplementation on Glucose and Lipid Metabolism in NIDDM. Diabetes.

[39] Zibaeenezhad M., Aghasadeghi K., Hakimi H., Yarmohammadi H., Nikaein F. (2016). The Effect of Walnut Oil Consumption on Blood Sugar in Patients with Diabetes Mellitus Type 2. Int. J. Endocrinol. Metab..

[40] Foster M., Petocz P., Caterson I.D., Samman S. (2013). Effects of zinc and α-linolenic acid supplementation on glycemia and lipidemia in women with type 2 diabetes mellitus: A randomized, double-blind, placebo-controlled trial. J. Diabetes Res. Clin. Metab..

[41] Jenkins D.J., Kendall C.W., Vuksan V., Faulkner D., Augustin L.S., Mitchell S., Ireland C., Srichaikul K., Mirrahimi A., Chiavaroli L. (2014). Effect of Lowering the Glycemic Load With Canola Oil on Glycemic Control and Cardiovascular Risk Factors: A Randomized Controlled Trial. Diabetes Care.

[42] Taylor C.G., Noto A.D., Stringer D.M., Froese S., Malcolmson L. (2010). Dietary Milled Flaxseed and Flaxseed Oil Improve N-3 Fatty Acid Status and Do Not Affect Glycemic Control in Individuals with Well-Controlled Type 2 Diabetes. J. Am. Nutr..

[43] Norris L.E., Collene A.L., Asp M.L., Hsu J.C., Liu L.-F., Richardson J.R., Li D., Bell D., Osei K., Jackson R.D. (2009). Comparison of dietary conjugated linoleic acid with safflower oil on body composition in obese postmenopausal women with type 2 diabetes mellitus1234. Am. J. Clin. Nutr..

[44] Barre D.E., Mizier-Barre K.A., Griscti O., Hafez K. (2008). High Dose Flaxseed Oil Supplementation May Affect Fasting Blood Serum Glucose Management in Human Type 2 Diabetics. J. Oleo Sci..

[45] Gerhard G.T., Ahmann A., Meeuws K., McMurry M.P., Duell P.B., Connor W.E. (2004). Effects of a low-fat diet compared with those of a high-monounsaturated fat diet on body weight, plasma lipids and lipoproteins, and glycemic control in type 2 diabetes. Am. J. Clin. Nutr..

[46] Brynes A.E., Edwards C.M., Jadhav A., Ghatei M.A., Bloom S.R., Frost G.S. (2000). Diet-induced change in fatty acid composition of plasma triacylglycerols is not associated with change in glucagon-like peptide 1 or insulin sensitivity in people with type 2 diabetes. Am. J. Clin. Nutr..

[47] Tsihlias E.B., Gibbs A.L., McBurney M.I., Wolever T.M. (2000). Comparison of high- and low-glycemic-index breakfast cereals with monounsaturated fat in the long-term dietary management of type 2 diabetes. Am. J. Clin. Nutr..

[48] Christiansen E., Schnider S., Palmvig B., Tauber-Lassen E., Pedersen O. (1997). Intake of a Diet High in Trans Monounsaturated Fatty Acids or Saturated Fatty Acids: Effects on postprandial insulinemia and glycemia in obese patients with NIDDM. Diabetes Care.

[49] Lerman-Garber I., Ichazo-Cerro S., Cardoso-Saldaña G., Posadas-Romero C., Zamora-Gonzalez J. (1994). Effect of a High-Monounsaturated Fat Diet Enriched With Avocado in NIDDM Patients. Diabetes Care.

[50] Heine R.J., Mulder C., Popp-Snijders C., Van Der Meer J., Van Der Veen E.A. (1989). Linoleic-acid-enriched diet: Long-term effects on serum lipoprotein and apolipoprotein concentrations and insulin sensitivity in noninsulin-dependent diabetic patients. Am. J. Clin. Nutr..

[51] Mohan V., Gayathri R., Lakshmipriya N., Anjana R.M., Spiegelman D., Jeevan R.G., Balasubramaniam K.K., Jayanthan M., Gopinath V., Divya S. (2018). Cashew Nut Consumption Increases HDL Cholesterol and Reduces Systolic Blood Pressure in Asian Indians with Type 2 Diabetes: A 12-Week Randomized Controlled Trial. J. Nutr..

[52] Sauder K.A., McCrea C.E., Ulbrecht J.S., Kris-Etherton P.M., West S.G., Kris-Ethertonb S.G.W.P.M. (2015). Effects of pistachios on the lipid/lipoprotein profile, glycemic control, inflammation, and endothelial function in type 2 diabetes: A randomized trial. Metab. Clin. Exp..

[53] Parham M., Heidari S., Khorramirad A., Hozoori M., Hosseinzadeh F., Bakhtyari L., Vafaeimanesh J. (2014). Effects of Pistachio Nut Supplementation on Blood Glucose in Patients with Type 2 Diabetes: A Randomized Crossover Trial. Diabetes Stud..

[54] Wien M., Oda K., Sabaté J. (2014). A randomized controlled trial to evaluate the effect of incorporating peanuts into an American Diabetes Association meal plan on the nutrient profile of the total diet and cardiometabolic parameters of adults with type 2 diabetes. Nutr. J..

[55] Kendall C., Esfahani A., Josse A., Augustin L., Vidgen E., Jenkins D. (2011). The glycemic effect of nut-enriched meals in healthy and diabetic subjects. Nutr. Metab. Cardiovasc. Dis..

[56] Bang H.O., Dyerberg J. (1972). Plasma lipids and lipoproteins in Greenlandic west coast Eskimos. Acta Med. Scand..

[57] Mozaffarian D., Wu J.H. (2011). Omega-3 fatty acids and cardiovascular disease: Effects on risk factors, molecular pathways, and clinical events. J. Am. Coll. Cardiol..

[58] Delany J.P., Vivian V.M., Snook J.T., Anderson P.A. (1990). Effects of fish oil on serum lipids in men during a controlled feeding trial. Am. J. Clin. Nutr..

[59] Saynor R., Verel D., Gillott T. (1984). The long-term effect of dietary supplementation with fish lipid concentrate on serum lipids, bleeding time, platelets and angina. Atherosclerosis.

[60] Akinkuolie A.O., Ngwa J.S., Meigs J.B., Djoussé L. (2011). Omega-3 polyunsaturated fatty acid and insulin sensitivity: A meta-analysis of randomized controlled trials. Clin. Nutr..

[61] Vessby B., Aro A., Skarfors E., Berglund L., Salminen I., Lithell H. (1994). The Risk to Develop NIDDM Is Related to the Fatty Acid Composition of the Serum Cholesterol Esters. Diabetes.

[62] Sacks F.M., Campos H. (2006). Polyunsaturated Fatty Acids, Inflammation, and Cardiovascular Disease: Time to Widen Our View of the Mechanisms. J. Clin. Endocrinol. Metab..

[63] Farvid M.S., Ding M., Pan A., Sun Q., Chiuve S.E., Steffen L.M., Willett W.C., Hu F.B. (2014). Dietary Linoleic Acid and Risk of Coronary Heart Disease: A Systematic Review and Meta-Analysis of Prospective Cohort Studies. Circulation.

[64] Warensjö E., Sundström J., Vessby B., Cederholm T., Risérus U. (2008). Markers of dietary fat quality and fatty acid desaturation as predictors of total and cardiovascular mortality: A population-based prospective study. Am. J. Clin. Nutr..

[65] Kim J.H., Kim Y., Kim Y.J., Park Y. (2016). Conjugated Linoleic Acid: Potential Health Benefits as a Functional Food Ingredient. Annu. Rev. Food Sci. Technol..

[66] Brouwer I.A., Wanders A.J., Katan M.B. (2013). Trans fatty acids and cardiovascular health: Research completed?. Eur. J. Clin. Nutr..

[67] Wu J.H.Y., Marklund M., Imamura F., Tintle N., Korat A.V.A., De Goede J., Zhou X., Yang W.-S., Otto M.C.D.O., Kröger J. (2017). Omega-6 fatty acid biomarkers and incident type 2 diabetes: Pooled analysis of individual-level data for 39 740 adults from 20 prospective cohort studies. Lancet Diabetes Endocrinol..

[68] Summers L.K.M., Fielding B.A., Bradshaw H.A., Ilic V., Beysen C., Clark M.L., Moore N.R., Frayn K.N. (2002). Substituting dietary saturated fat with polyunsaturated fat changes abdominal fat distribution and improves insulin sensitivity. Diabetologia.

[69] Ros E. (2015). Nuts and CVD. Br. J. Nutr..

[70] Luo C., Zhang Y., Ding Y., Shan Z., Chen S., Yu M., Hu F.B., Liu L. (2014). Nut consumption and risk of type 2 diabetes, cardiovascular disease, and all-cause mortality: A systematic review and meta-analysis. Am. J. Clin. Nutr..

[71] Jiang R., Manson J.E., Stampfer M.J., Liu S., Willett W.C., Hu F.B. (2002). Nut and Peanut Butter Consumption and Risk of Type 2 Diabetes in Women. JAMA.

[72] Salas-Salvadó J., Bulló M., Babio N., Martínez-González M.Á., Ibarrola-Jurado N., Basora J., Estruch R., Covas M.I., Corella D., Arós F. (2011). Reduction in the incidence of type 2 diabetes with the Mediterranean diet: Results of the PREDIMED-Reus nutrition intervention randomized trial. Diabetes Care.

[73] Jovanovski E., Li D., Ho H.V.T., Djedovic V., Marques A.D.C.R., Shishtar E., Mejia S.B., Sievenpiper J.L., De Souza R.J., Duvnjak L. (2017). The effect of alpha-linolenic acid on glycemic control in individuals with type 2 diabetes: A systematic review and meta-analysis of randomized controlled clinical trials. Medicine.

[74] Vessby B., Uusitupa M., Hermansen K., Riccardi G., Rivellese A.A., Tapsell L.C., Nälsén C., Berglund L., Louheranta A., Rasmussen B.M. (2001). Substituting dietary saturated for monounsaturated fat impairs insulin sensitivity in healthy men and women: The KANWU study. Diabetologia.

